# OpenWMB: An open-source and automated working memory task battery for OpenSesame

**DOI:** 10.3758/s13428-024-02397-1

**Published:** 2024-04-04

**Authors:** Fábio Monteiro, Letícia Botan Nascimento, José Leitão, Eduardo J. R. Santos, Paulo Rodrigues, Isabel M. Santos, Fátima Simões, Carla S. Nascimento

**Affiliations:** 1 CINEICC - Center for Research in Neuropsychology and Cognitive Behavioral Intervention, Faculty of Psychology and Educational Sciences, University of Coimbra, Coimbra, Portugal; 2 ChronoCog – Laboratory for Chronopsychology and Cognitive Systems, Faculty of Psychology and Educational Sciences, University of Coimbra, Coimbra, Portugal; 3 Department of Psychology and Education, University of Beira Interior, Covilhã, Portugal; 4 William James Center for Research, University of Aveiro, Aveiro, Portugal; 5 Faculty of Psychology and Educational Sciences, University of Coimbra, Coimbra, Portugal; 6 SHERU – Sport, Health & Exercise Research Unit, Polytechnic Institute of Castelo Branco, Castelo Branco, Portugal; 7 Center for Research in Education and Psychology, University of Évora, Évora, Portugal

**Keywords:** Working memory capacity, Psychometry, Computerised cognitive assessment, Methodology, OpenSesame

## Abstract

Working memory capacity (WMC) has been measured with a plethora of cognitive tasks. Several preeminent automated batteries of working memory (WM) tasks have been developed recently. However, despite all their advantages, most batteries were programmed in paid platforms and/or only included a single WM paradigm. To address these issues, we developed the OpenWMB, an automated battery comprising seven tasks from three distinct paradigms (complex spans, updating tasks, and binding tasks) that tap into several functional aspects of WM (simultaneous storage and processing, updating, and binding). The battery runs on open-source software (OpenSesame) and is freely available online in a ready-to-download format. The OpenWMB possesses flexible features and includes a data processing script (that converts data into a format ready for statistical analysis). The instrument is available in Portuguese and English. However, we only assessed the psychometric properties of the former version. The Portuguese version presented good internal consistency and considerable internal and predictive validity: all tasks loaded into a single factor. Additionally, the WMC estimate was strongly correlated with a fluid intelligence factor. This study also tried to contribute to the ongoing debate regarding the best method to assess WMC. We computed a permutation analysis to compare the amount of variance shared between a fluid intelligence factor and (1) each WM task, (2) homogenous WMC factors (based on multiple tasks from the same paradigm), and (3) heterogeneous WMC factors (derived from triplets of tasks from different paradigms). Our results suggested that heterogeneous factors provided the best estimates of WMC.

## Introduction

Working memory (WM) is a limited-capacity system that temporarily stores, manipulates, and retrieves information necessary for ongoing cognitive processes (Baddeley, 2012; Unsworth et al., 2009; Wilhelm et al., 2013). The underlying structure of WM remains a subject of broad debate. Some authors propose that WM can be fractionated into separable functional aspects (simultaneous storage and processing of information, updating of mental representations, and binding of information elements into structures) (Ecker et al., 2010; Himi et al., 2019; Oberauer et al., 2003) and specific content domains (verbal-numeric and visuospatial) (Oberauer et al., 2000; Waris et al., 2017), while others view this process as a global cognitive resource (Engle et al., 1999; Kane et al., 2004).

WM has been associated with a multitude of high-level cognitive abilities, such as rationality (Burgoyne et al., 2023), fluid intelligence (*Gf*) (Felez-Nobrega et al., 2018; Rey-Mermet et al., 2019), reading comprehension (Pham & Hasson, 2014), speech production (Herman et al., 2013), drawing capacity (Trojano et al., 2004), and arithmetical abilities (Hubber et al., 2014). Moreover, it influences different aspects of everyday life — e.g., multi-tasking (Hambrick et al., 2010), and resisting the continued influence of misinformation (Brydges et al., 2018). Additionally, a decline in WM has been linked with several cognitive deficits and clinical pathologies, including attention deficit disorder (Kofler et al., 2019) and schizophrenia (Braun et al., 2021).

Considering the crucial role of WM in human cognition, the development of universally accessible and well-validated tools to measure individual differences in WM capacity (WMC) is critical (Redick et al., 2012). While recently several notable examples of automated batteries of WM tasks have been freely made available online (Foster et al., 2015; Lewandowsky et al., 2010; Ma et al., 2017; Oswald et al., 2015; Stone & Towse, 2015; Unsworth et al., 2005, 2009), many were programmed in paid platforms making them inaccessible to researchers who do not have access to such resources. Furthermore, these batteries are still only available in certain languages — for instance, none of these instruments includes a Portuguese version. To address these needs, we developed the OpenWMB, an open-source and automated battery that contains multiple tasks from three WM paradigms (complex spans, updating tasks, and binding tasks) and is available in both Portuguese and English. In this article, we offer a comprehensive description of the features of the battery and provide a step-by-step guide on how to install and run the OpenWMB, and how to process data collected with this instrument. We also report the validation study that was conducted to assess the psychometric proprieties of the Portuguese version of the OpenWMB. Additionally, we used the data collected in this study to evaluate which method yielded the best estimate of WMC among (1) single WM tasks, (2) homogenous WMC factors (based on multiple tasks from the same paradigm), and (3) heterogeneous WMC factors (derived from triplets of tasks from different paradigms). To test this, we compared the amount of variance shared between a *Gf* factor and all possible combinations of single WM tasks, homogenous, and heterogeneous WMC factors.

Due to its intangible nature, WM is evaluated with tasks that measure WMC indirectly (Schmiedek et al., 2014). In the past years, three paradigms have been regularly administrated to assess WMC: complex spans (Daneman & Carpenter, 1980; Turner & Engle, 1989), updating tasks (Kirchner, 1958; Salthouse et al., 1991), and binding tasks (Quinette et al., 2006; Wilhelm et al., 2013). Complex spans are dual tasks that require keeping information in an active state while completing a secondary task (Redick et al., 2012; Unsworth et al., 2009). Updating tasks involve continuously refreshing mental representations (Ecker et al., 2010). In binding tasks, the participants need to link several characteristics of the stimuli (e.g., position and verbal content) to build structures and establish new relationships (Oberauer et al., 2003; Wilhelm et al., 2013).

Several studies suggested that these three classes of WM tasks are reliable and valid measures of WMC. They present good internal consistency (Schmiedek et al., 2009; Wilhelm et al., 2013) and temporal stability (Redick et al., 2012; Soveri et al., 2018). They also present good predictive validity: unlike cognitive tasks that only encompass storage demands (such as simple spans), WM paradigms predict high-level cognitive abilities such as *Gf* (Engle et al., 1999; Kane et al., 2004; Wilhelm et al., 2013). Furthermore, there is some evidence that suggests that even though these paradigms present different structures and requirements they measure the same underlying construct. Several studies (Lewandowsky et al., 2010; Schmiedek et al., 2009, 2014; Waris et al., 2017; Wilhelm et al., 2013) suggested that complex spans, updating tasks, and binding tasks presented high loadings on general WMC factors, which is evidence of convergent validity. However, these findings are not consensual. For instance, Jaeggi et al. (2010) and Kane et al. (2007) found weak correlations between complex spans and the n-back task (which is classified as an updating task according to the definitions used in this article).

The differences between the results of these studies may be explained by the use of different analytical techniques to assess the relationship between different classes of WM tasks: Lewandowsky et al. (2010), Schmiedek et al. (2009, 2014), Waris et al. (2017), and Wilhelm et al. (2013) used factorial analyses — a statistical procedure that extracts common variance shared by the observed variables to identify latent factors — to assess the aforementioned relationship, while Jaeggi et al. (2010) and Kane et al. (2007) employed correlations (a method that estimates the relationship between two variables at the observed level) to evaluate this phenomenon. Thus, using different analytical procedures may have changed the intensity and directions of the relationships between the WM tasks which may explain the different conclusions regarding this issue (Schmiedek et al., 2009).

There are other technical aspects that need to be considered during the administration of WM tasks because they may also lead to different reliability estimates and change the nature of the measurements of these tasks. Among these aspects, we would like to highlight the use of different scoring techniques (e.g., partial-credit scoring vs. absolute scoring) (Conway et al., 2005), administration methods (computer-paced, experimenter-paced, or participant-paced) (Bailey, 2012; Friedman & Miyake, 2004), and presentation orders (presenting blocks of trials with different sizes in ascending order vs. presenting blocks of trials with different sizes in random order) (Unsworth et al., 2005). For instance, Friedman and Miyake (2004) found that an experimenter-paced version of a complex span presented a stronger correlation with reading comprehension measures than a participant-paced version of the same task. These authors argued that the participants may have used different strategies to complete the two versions of the complex span and that these differences may have changed the nature of what the task was actually measuring. A thorough review of the consequences of these technical issues is beyond the scope of this article. However, see Conway et al. (2005) and Friedman and Miyake (2004) for an extensive discussion regarding these topics.

Another important aspect that needs to be considered in investigations that seek to assess WMC is the number of tasks that need to be employed to achieve the best measurement of this construct. Given the good reliability indexes and the established validity of most WM tasks, depending on the objectives of the investigation, the administration of a single test to estimate WMC is feasible (Wilhelm et al., 2013). However, despite their good psychometric proprieties, no WM task is a perfect measure of WMC. Every one of these tests taps into variance caused by individual differences in WMC but also encapsulates variance produced by the idiosyncratic features of the paradigm and the task (e.g., the structure of the task or the content domain of the stimuli), and measurement error (Engle et al., 1999; Foster et al., 2015). Applying a single task makes it difficult to separate variance caused by individual differences in WMC from variance caused by the specific features of the task (Schmiedek et al., 2014) — this can be particularly problematic for studies that need to extract “pure” WMC measurements. Furthermore, it is unlikely that a single task will cover the wide range of functions and domains attributed to WM (Lewandowsky et al., 2010).

To bypass this issues, if the time and resources are available, several authors recommend using more than one task to measure WMC and estimate this construct based on a composite score (computed by averaging the performance on all administrated tasks) (Schmiedek et al., 2014) or a latent factor (Conway et al., 2005; Foster et al., 2015; Wilhelm et al., 2013). Between the two approaches, only factorial analysis separates construct variance from paradigm and task-specific variance and measurement error (Schmiedek et al., 2009). Previous research suggested that there are some benefits in extracting the latent factor(s) from tasks from different paradigms because a factor based on tasks from the same paradigm will likely group paradigm-specific and WMC variance together (Lewandowsky et al., 2010; Redick et al., 2012). On the other hand, deriving WMC factors from several tasks from multiple paradigms that tap into different content domains and functional aspects ascribed to WM will significantly reduce bias in the interpretation of the derived factor(s) while also partialling out construct-irrelevant variance (Oswald et al., 2015; Schmiedek et al., 2014; Waris et al., 2017).

However, there is still no definitive answer about the best method to assess WMC (Kane et al., 2004; Schmiedek et al., 2009). Task selection should always consider the particularities of each investigation. Different combinations of tasks may be appropriate depending on whether researchers are only interested in assessing some dimensions of WM — e.g., binding tasks are particularly well suited to assess binding mechanisms (Oberauer et al., 2003) —, the characteristics of the target population (e.g., some tasks are more appropriate for children than adults) (Scharfen et al., 2018), or the time available to implement the experiment (e.g., administrating complex spans usually requires more time than employing updating or binding tasks) (Wilhelm et al., 2013).

In fact, administrating some WM tasks can be quite time-consuming. This may be one of the main reasons behind the implementation of a single task in several studies (Foster et al., 2015; Ma et al., 2017). To tackle this issue, several automated batteries of WM tasks have been developed in recent years. Automated WM batteries present several advantages in comparison to their pen-and-paper counterparts. They are usually less time-consuming, present automatic scoring, can randomize the presentation order of the trials, and can vary the number of trials in each administration (Oswald et al., 2015; Redick et al., 2012). Above all else, these batteries may be administered in group settings, allowing data collection from multiple participants simultaneously (Ma et al., 2017; Stone & Towse, 2015).

Currently, researchers have at their disposal some outstanding examples of automated WM batteries available online (Foster et al., 2015; Lewandowsky et al., 2010; Ma et al., 2017; Oswald et al., 2015; Stone & Towse, 2015; Unsworth et al., 2005, 2009). Most of these batteries were subjected to extensive validation. Some include tasks from different paradigms and possess flexible features — e.g., users can choose to administrate only a portion of the tasks and determine the number of trials they want to run in each task. Additionally, some of these instruments are available in multiple languages (e.g., English (Foster et al., 2015; Lewandowsky et al., 2010; Oswald et al., 2015; Stone & Towse, 2015), Chinese (Lewandowsky et al., 2010), and Spanish (Felez-Nobrega et al., 2018)) which is vital to establish the psychometric proprieties of these tools across different cultures — however, to the best of our knowledge, currently there is no automated battery of WM tasks freely accessible in a ready-to-download format for the Portuguese population. Despite all their notable advantages, the available automated WM batteries also present some drawbacks: most instruments were programmed in paid platforms, like Matlab or E-Prime, which means that researchers must have institutional access or purchase a commercial license for this software to use these batteries (which may not be an option for some researchers, especially PhD students). Additionally, most of them only include complex spans which may reduce their utility for investigators who need to separate unique WMC variance from paradigm-specific variance.

To help solve these gaps, we developed the OpenWMB, an automated battery of WM tasks that is entirely open-source and possesses several flexible features. The OpenWMB was programmed in OpenSesame (Mathôt et al., 2012) using Python and OpenSesame scripting. The battery can be downloaded from the GitHub repository associated with the webpage https://zenodo.org/doi/10.5281/zenodo.10600494 — to access this repository you will need to locate and click on the GitHub URL that is presented on the Zenodo page.

The instrument includes three complex spans (reading span, operation span, and symmetry span), two updating tasks (n-back task and memory updating task), and two binding tasks (binding and maintenance task and multimodal span). We selected these tasks because they presented high loadings on both general and functional or content-specific factors of WMC in previous studies (Kane et al., 2004; Schmiedek et al., 2009; Unsworth et al., 2009; Wilhelm et al., 2013). Also, they presented large correlations with *Gf* tasks (Oswald et al., 2015; Schmiedek et al., 2014). We included three paradigms in our battery because we believe they may help to account for a larger proportion of WMC variance. After all, it is probable that each paradigm taps into different functional aspects and content domains of WM (Oberauer et al., 2000, 2003). Thus, complex spans were included to capture variance prompted by the capacity to store and process information simultaneously; updating tasks were selected to evaluate the ability to continuously refresh mental representations; and binding tasks were chosen as measures of the capacity to link characteristics of information to form new structures.

We programmed three complex spans with stimuli from different content domains because some studies suggested that a small but non-negligible portion of the variance in these tasks was content-specific (verbal/numerical vs. spatial) (Kane et al., 2004; Oberauer et al., 2000). On the other hand, we only selected two updating tasks and two binding tasks because most investigations suggested that the ability to continuously update mental representations and the capacity to bind characteristics of information to form new structures are domain-general (Baddeley, 2000; Oberauer et al., 2003; Waris et al., 2017) — although some studies disagree with this premise (Nee et al., 2013). Even though a single updating task and a single binding task may have sufficed to account for the variance shared between these two paradigms and the complex spans, we included two updating tasks and two binding measures to control for task-specific variance. Thus, the OpenWMB includes heterogeneous measures that produce a reliable and valid general estimate of WMC by tapping both into its functional aspects (simultaneous storage and processing, updating, and binding abilities) and content domains (verbal, numeric, and spatial) while reducing paradigm-specific and task-specific variance.

The OpenWMB has some flexible features that can be implemented without any programming knowledge. For instance, users can choose only to administrate a portion of the tasks or just a single task. The battery is suitable for group testing, is entirely computer-paced, has embedded instructions for each task, and has automatic scoring. Additionally, the OpenWMB includes a data processing script that converts all data collected into an easily interpretable format that is ready for data analysis (in platforms like R or SPSS). A detailed description of the features of the instrument is presented in the Appendix.

The OpenWMB is available in Portuguese and English. However, we only assessed the psychometric properties of the Portuguese version — all analyses presented in this paper concern this version of the battery. All the information and caveats regarding the English version are presented in the last subsection of the Appendix. To assess the psychometric properties of the Portuguese version of the instrument, we administered the WM tasks included in the OpenWMB and three *Gf* measures to a sample of Portuguese adults. This study assessed the internal consistency, convergent validity, and predictive validity of the OpenWMB. The internal consistency of the battery was determined by calculating Cronbach’s alpha (*α*) and McDonald’s omega (*ω*) for each task. Its convergent validity was established by examining the relationships between the WM tasks at a latent level. To determine the predictive validity of the OpenWMB, we assessed the magnitude of the correlation between a latent factor derived from all WM tasks included in the battery and a latent factor extracted from the three *Gf* measures. Additionally, this study tested which method provided the best estimate of WMC among (1) single WM tasks, (2) homogenous WMC factors (based on multiple tasks from the same paradigm), and (3) heterogeneous WMC factors (derived from triplets of tasks from different paradigms). For this purpose, we compared the amount of variance shared between the *Gf* factor and all possible combinations of single WM tasks, homogenous, and heterogeneous WMC factors. A detailed account of the validation study will be presented in the following sections of the article.

## Method

### Participants

One hundred and sixty-nine individuals participated in a single experimental session. The participants were recruited through e-mail and direct contact (personal referrals and direct approaches at the campuses). The participants did not receive any payment or other benefits for participating in the study. Participation in this study was restricted to Portuguese citizens aged 18 to 35 years who held at least a high school degree. Data from five participants were discarded because they did not meet the inclusion criteria (three participants did not possess Portuguese citizenship, and another two were older than 35 years). One participant scored 0 in more than one cognitive task and was excluded. Another participant was classified as a multivariate outlier and was excluded from further analyses. Thus, our analyses were based on data from 162 participants (52 male; age range = 18-33 years, mean age = 22.25, *SD* = 4.12). The sample had a diverse academic background. Approximately 67.9% of the participants were university students — 54.9% were undergraduate students, and 13% were postgraduate students. The rest of the sample consisted of participants who had already completed their studies. 9.3% of the participants held a high school degree, while 22.8% completed at least one higher education degree.

### Apparatus, design, and procedure

The tasks were programmed in OpenSesame (version 3.3.11) (Mathôt et al., 2012). The Mousetrap plugin for OpenSesame (Kieslich & Henninger, 2017) was used to track mouse movements in the symmetry and multimodal spans. The participants completed the WM and *Gf* tasks in a single session. Up to 12 participants were tested simultaneously in a soundproof room. The test session took approximately 2h20. In the middle of the session, the participants were granted a 20-minute break during which they were offered snacks (water, juice, fruit, cookies, and sandwiches). General instructions were provided at the start of the session. Specific instructions for each task were embedded in the program and were presented on the computer screen. Practice trials were completed before each task.

The order of the tasks was counterbalanced between-participants with a Latin square design (a form of partial counterbalancing) (Grant, 1948). The order of the tasks was controlled by an algorithm embedded in our program that ensured that all tasks were presented an equal number of times in each position across participants.

### Materials

#### Complex spans

*Reading span* (Daneman & Carpenter, 1980). Several blocks^1^ of interleaved sentences and letters were presented in this task. The participants were required to read sentences and determine if they presented syntactic errors while trying to remember the sequence of presented letters. At the end of each block, the participants had to recall the to-be-remembered letters in the same order they were presented.

The processing and memorization portions of the task were presented on different canvases — the same paradigm was used in the operation and symmetry spans. Each trial^1^ began with the presentation of a fixation dot (for 500 ms). Then, a sentence was displayed. Each sentence had 11 or 12 words and 45 to 57 characters. The participants were instructed to press the key ‘1’ when they thought the sentence did not present a syntactic error and the key ‘2’ when they thought the sentence contained a syntactic error. Half of the sentences presented correct syntax. Syntactically incorrect sentences had a single error.

The time that each participant had to read a sentence and state if it presented a syntactic error was adjusted for each participant — a calibration task was completed before the reading span. In this task, the participants read 20 sentences and signaled if they presented syntactic errors or not. The time each participant had to process each sentence in the reading span was the same as his mean reaction time in the calibration task + 2.5 standard deviations (Unsworth et al., 2009). Several authors suggested that constraining the time each participant has to process sentences reduces the influence of different rehearsal strategies, which leads to a purer estimate of the simultaneous storage and processing capacity of the participants (Redick et al., 2012; Unsworth et al., 2005).

After each sentence, a single letter was displayed for 1000 ms. The sequences of to-be-remembered letters were randomly generated by an algorithm. Thus, the sequences of letters changed in each administration of the task. This enabled us to control possible order effects. Additionally, our algorithm ensured that each letter only appeared a single time in each block of trials. The participants were asked to memorize the letters in the same order they were presented.

At the end of each block, they were required to type the to-be-remembered letters on a response box. There was no time constraint for the recall of the to-be-remembered letters. To-be-remembered letters were lowercase monosyllabic — in Portuguese — consonants (b, c, d, f, g, p, q, t, v, x, z). We selected these letters to ensure that all to-be-remembered letters had a similar rehearsal cost. We decided to use letters instead of words as to-be-remembered items because word knowledge may account for some of the variance shared between measures of higher-order cognitive functions and span tasks that use words as to-be-remembered items (Engle et al., 1990). At the end of each block, the participants received feedback regarding the number of correctly recalled to-be-remembered letters and the number of correct responses in the processing portion of the task.

Block sizes ranged from two to six (total number of trials: 60) and were presented in ascending order. Three blocks were administrated per set size. This task also included two practice blocks, each containing two sentences and two to-be-remembered letters.

**Fig. 1:**
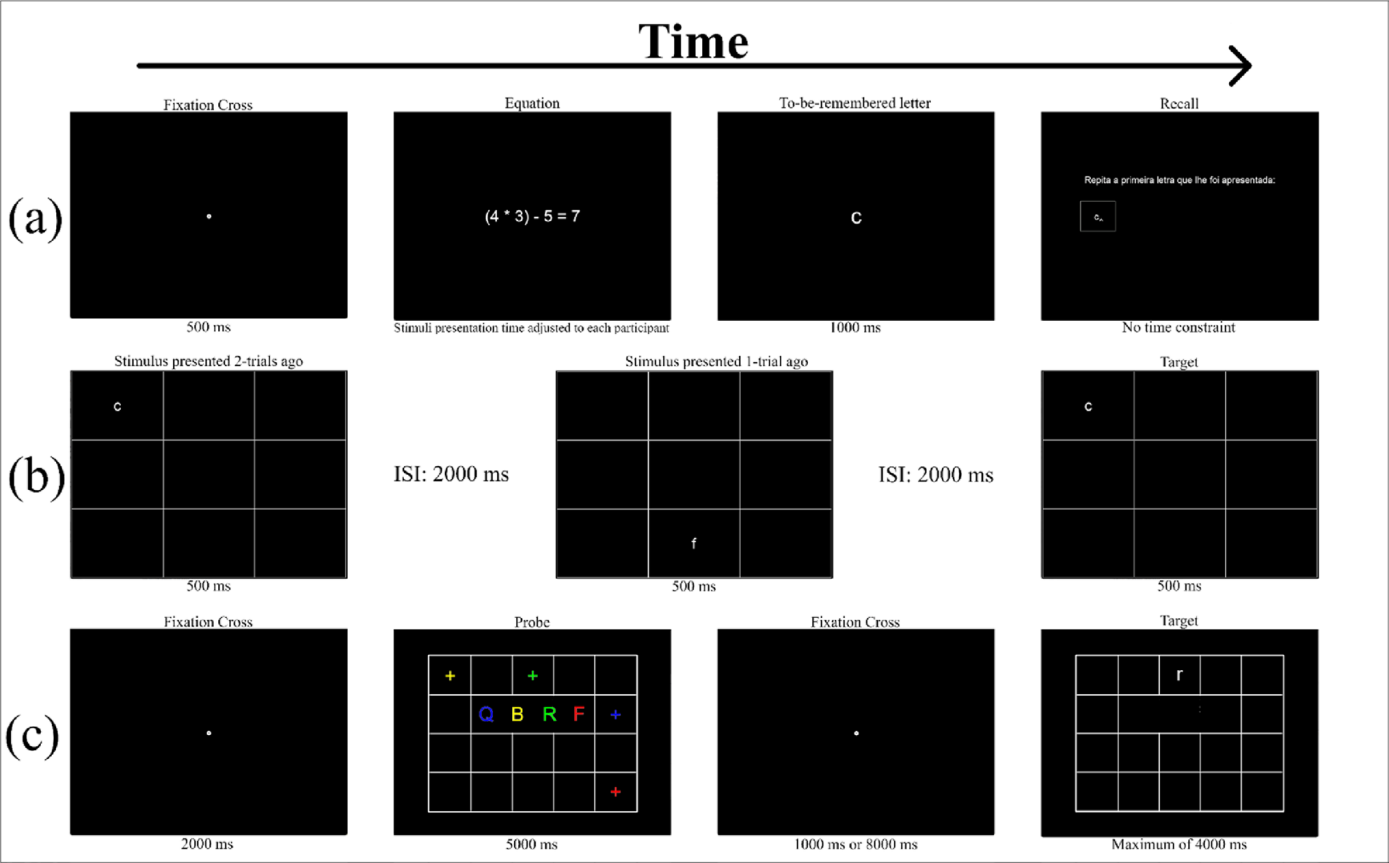
Schematic representation of some of the tasks included in the OpenWMB. (**a**) Operation span; (**b**) n-back task; (**c**) binding and maintenance task; ISI, interstimulus interval

*Operation span* (Turner & Engle, 1989). Like in the reading span, the participants had to perform a processing task while simultaneously holding unrelated information in memory. A schematic overview of the operation span is presented in Fig. 1a. In this case, they had to solve several blocks of equations and indicate if the result presented by our program was correct while trying to remember a sequence of letters. At the end of each block, they had to recall the to-be-remembered letters.

A fixation dot was presented at the beginning of each trial (for 500 ms). Then, an equation was displayed. Each equation had the same structure (e.g., (5 * 2) + 4 = 14): The first term was a multiplication or a division (presented inside brackets) between two integers between 1 and 9. The result of this operation was a positive integer between 1 and 20. The second term was a sum or a subtraction between the resulting number of the first term and an integer ranging from 1 to 9. The result of the second term was also comprehended between 1 and 20 (Pardo-Vazquez & Fernandez-Rey, 2008). The participants had to press the key ‘1’ if they considered that the result of the equation was correct or the key ‘2’ if they thought that the result was incorrect. Half of the equations presented correct results. In trials with incorrect results, the difference between the proposed and correct results was never higher than 2. The time each participant had to solve an equation and state if the result was correct or incorrect was adjusted for each individual with a method similar to the one used for the reading span. In this calibration task, the participants were asked to solve 20 equations (Unsworth et al., 2005). This version of the operation span comprised the same proportion of (1) equations with a multiplication on the first term and an addition on the second term and a correct result, (2) equations with a multiplication on the first term and an addition on the second term and an incorrect result, (3) equations with a multiplication on the first term and a subtraction on the second term and a correct result, (4) equations with a multiplication on the first term and a subtraction on the second term and an incorrect result, (5) equations with a division on the first term and an addition on the second term and a correct result, (6) equations with a division on the first term and an addition on the second term and an incorrect result, (7) equations with a division on the first term and subtraction on the second term and a correct result, and (8) equations with a division on the first term and subtraction on the second term and an incorrect result. The presentation order of the equations was counterbalanced with a Latin square design (Grant, 1948) that ensured that each type of equation was presented an equal number of times in each position to every participant.

After each equation, a single random letter was displayed for 1000 ms (each letter only appeared once in each block). Sequences of to-be-remembered letters were randomly generated by an algorithm and changed in each administration. The participants were asked to memorize this set of letters in the same order they were presented.

At the end of each block, they typed the to-be-remembered letters on a response box. No time constraint was imposed on the recall of the to-be-remembered letters. The list of to-be-remembered letters was the same as the reading span (b, c, d, f, g, p, q, t, v, x, z). At the end of each block, feedback was provided regarding the amount of correctly recalled to-be-remembered letters and the number of correct responses in the processing portion of the task.

Block sizes ranged from two to six (total number of trials: 60) and were presented in ascending order. Three blocks were administrated per set size. This task also included two practice blocks, each containing two equations and two to-be-remembered letters.

*Symmetry span* (Kane et al., 2004). Like in the other two complex spans, each trial of this task included interleaved processing and memory sub-tasks. In the processing segment, the participants had to indicate whether 8 × 8 matrices of black and white squares were symmetrical. After each matrix, the participants had to memorize the position of a red square presented on a 4 × 4 grid. They were asked to recall the position of all red squares in the same order they were presented at the end of each block of trials.

The presentation of a fixation dot for 500 ms signaled the beginning of each trial. Then, an 8 × 8 matrix of black and white squares was presented. The participants had to decide whether the pattern of black and white squares was symmetrical along its vertical axis (the left half of the matrix had to mirror the right half). The participants were instructed to press the key ‘1’ when they thought the matrices were symmetrical or press the key ‘2’ when they considered this was not the case. Half of the matrices were symmetrical. On dissymmetrical trials, only a single square was different on the right and the left halves of the matrices. Like in the reading span and the operation span, the time each participant had to complete the processing portion of the task was individually adjusted. In this case, the participants were asked whether 20 matrices were symmetrical or not in the calibration task (Unsworth et al., 2009).

After each matrix — in the memory portion of the symmetry task — a 4 × 4 grid containing a single red square was presented for 1000 ms. The sequences of red squares were randomly generated by an algorithm and changed in each administration. The participants were instructed to memorize the positions of the red squares.

At the end of each block of trials, they had to recall the sequence of red squares and type them on an empty 4 × 4 grid with the mouse. Each time a red square was typed, the respective cell was highlighted (it turned red for 500 ms). No time constraint was imposed on the recall of the positions of the red squares. At the end of each block, feedback was provided regarding the number of correctly recalled to-be-remembered red squares and the number of correct decisions in the symmetrical/dissymmetrical sub-task.

Set sizes ranged from two to six (total number of trials: 60) and were presented in ascending order. Three blocks were administrated per set size. This task also included two practice blocks, each containing two similar/dissimilar matrices and two to-be-remembered red squares.

#### Updating tasks

*N-back task* (Kirchner, 1958; Schmiedek et al., 2009). A graphical overview of this task is shown in Fig. 1b. A series of letters was continuously presented on a 3 × 3 grid. In this version of the n-back task, the participants were required to constantly update both verbal and spatial contents of the stimuli. The participants had to assess whether the stimulus presented on the grid was identical to the stimulus presented two trials ago — the same letter presented in the same cell of the grid. The participants were asked to press the key ‘m’ if this condition was met. When the condition was not met, the participants did not need to press any key. Every time the key ‘m’ was pressed, the cell in which the stimulus was presented was highlighted (the borders of the cell turned red) to let the participants know that the computer collected their response.

The n-back task included a practice block and a test block. The practice block included eight trials, and the test block contained 38. However, the first two trials of each block were preparatory because they had no reference stimulus to be compared with. Thus, only six trials of the practice block and 36 trials of the test block were scored. In both blocks, 1/3 of the trials were targets — trials in which the presented stimulus was the same as the one presented two trials ago. On the other 2/3 of the trials, there was a mismatch between the position and/or the verbal content of the presented stimulus and the stimulus presented two trials ago.

The list of to-be-updated letters was the same one used in the reading and operation spans (b, c, d, f, g, p, q, t, v, x, z). These ensured that the rehearsal and updating costs of all stimuli were identical. The sequence of stimuli presented in each administration of the n-back tasks was randomly generated by an algorithm and changed in each task administration. Furthermore, the n-back task was completely computer-paced.

The beginning of each block of trials was signaled by a fixation dot presented for 500 ms. Then, each letter was presented for 500 ms. When a letter disappeared, there was an interstimulus interval (ISI) of 2000 ms before the following letter was presented. The participants could type their response when the to-be-updated letter was presented or during the ISI. At the end of each block, the participants received feedback regarding the number of trials in which they correctly identified the target and non-target trials.

*Memory updating task* (Salthouse et al., 1991; Schmiedek et al., 2009). In this task, the participants had to continuously update the three digits presented at the beginning of the trial.

A fixation dot was presented at the beginning of each trial for 500 ms. Then, two rows with three frames each were displayed. A single-digit number was presented in each frame of the first row for 2000 ms. The participants were asked to memorize these digits. Then, a continuous sequence of subtractions and sums was presented in the second row of frames. The participants had to continuously update the numbers presented at the beginning of the trial by applying the mathematical operations (e.g., ‘+4’ or ‘-7’) presented in the corresponding frame of the second row and memorize the updated numbers. A single row with three empty frames was displayed when all updating operations were presented. The participants were requested to use the keyboard to type the updated digits in the corresponding frames. No time constraint was imposed in this segment of the task.

Each updating operation ranged from ‘-8’ to ‘+8’ and was displayed for 2000 ms (there was a 500 ms interval between each pair of operations). The results of all operations (both intermediate and final) were comprised between 1 and 9. In each trial, two non-consecutive updating operations were displayed in each frame of the second row. Thus, six updating operations were presented in each trial. Half of the operations were sums, and half were subtractions. All participants completed the same updating sequences. However, the sequences were presented in random order to prevent order effects.

This task consisted of a practice block and a test block. The participants had to complete two trials on the practice block and 12 trials in the test block. At the end of each trial, the participants received feedback regarding the number of digits they were able to successfully update — the correctly updated digits were also presented on the feedback screen. They also received feedback about the total number of correctly updated digits at the end of each block (a maximum of six digits in the practice block and 36 digits in the test block).

#### Binding tasks

*Binding and maintenance task* (Quinette et al., 2006). In this task, the participants had to bind verbal and spatial characteristics of the stimuli and keep this association in mind for varying periods. A schematic representation of the binding and maintenance task is presented in Fig. 1c.

Each trial began with a fixation dot presented for 2000 ms. Then, a 5 × 4 grid with four colored (red, yellow, blue, and green) uppercase letters at its center was displayed for 5000 ms. This grid also contained four crosses with matching colors placed randomly in the remaining 17 squares. The participants were requested to associate and bind each colored letter with the location of the cross with a matching color. Next, the grid with colored letters and crosses was replaced by a white fixation dot. This fixation dot was presented for 1000 ms in half of the trials and 8000 ms in the other half. The participants were expected to maintain the association between the colored letters and the matching crosses while this fixation dot was presented. A grid with a single white lowercase letter was shown in the last segment of each trial. The participants had 4000 ms to indicate if the position of the white letter matched the position of the cross with the same color as the corresponding letter in the first grid. The participants were instructed to press the key ‘1’ when the position of the white letter was the same as the cross with the same color as the corresponding letter in the first grid or the key ‘2’ when this condition was not met. There was a match between the position of the white letter and the position of the corresponding colored cross in half of the trials.

This version of the binding and maintenance task was completely computer-paced. The task included a practice block with four trials and a test block with 16 trials. A quarter of the trials in each block corresponded to (1) matching trials with a 1000 ms interval between the first and second grid, (2) mismatching trials with a 1000 ms interval between the first and second grid, (3) matching trials with an 8000 ms interval between the first and second grid, and (4) mismatching trials with an 8000 ms interval between the first and second grid. All participants completed the same trials — however, the trials were presented in random order. At the end of the practice and test blocks, the participants received feedback regarding the number of trials in which they correctly signaled whether the white letter was in the same position as the matching colored cross or not.

*Multimodal span* (Quinette et al., 2006). The participants were required to replicate several sequences of letters presented on a grid. Each trial began with a fixation dot displayed for 500 ms. Then, a series of letters was displayed sequentially on a 4 × 4 grid. Each letter was presented for 1000 ms (ISI: 1000 ms). After the presentation of each sequence, an empty 4 × 4 grid appeared. The participants tried to reproduce the presented sequences on the empty grid by typing the letters on the cells in which they were presented.

To type each letter, the participants were instructed first to use the mouse to select a cell — the borders of the cells turned red when they were pressed — and then to type the letter using the keyboard (the letter appeared for 500 ms inside the cell after it was typed). No time constraint was imposed when the participants were trying to replicate the sequence.

Each sequence was randomly generated by an algorithm. Thus, sequences changed in each administration of the task. Like in the other WM tasks, the set of letters used in this task was restricted to lowercase monosyllabic (in Portuguese) consonants — b, c, d, f, g, p, q, t, v, x, z. Each letter was only presented once in each sequence. Additionally, letters could not be presented in the same position twice in the same sequence.

The multimodal span included a practice block and a test block. In both blocks, the first sequence had a length of three elements (three letters presented sequentially in three different positions). If the participants were able to reproduce a sequence correctly — every letter needed to be typed in the correct positions in the same order that they were presented — the computer generated a new sequence with the same length as the previous sequence + 1 element. Sequence lengths ranged from three to 11. The test block was terminated if the participants were not able to replicate a sequence with a given length in two consecutive trials or if the participants were able to reproduce a sequence with a length of 11 elements. The practice block included sequences with lengths of three and four elements. In this block, the participants were allowed to complete all trials even if they were not able to replicate a sequence with a given length in two consecutive trials. In both practice and experimental blocks, feedback was provided at the end of each trial, informing participants if they were able to replicate the last presented sequence correctly.

#### Gf tasks

*Letter series* (Schrepp, 1999; Simon & Kotovsky, 1963)*.* This task was used to assess verbal inductive reasoning. In each trial, a series of letters (e.g., ‘abmcdmefmghm’) that followed some unstated logical pattern was presented — in this example, the series can be broken down into segments of three letters (e.g., ‘abm’, ‘cdm’). The first two letters of each segment move along the alphabet (‘ab’, ‘cd’), while the third letter is kept constant (‘m’). The participants had to identify the logical pattern, guess the next three letters of the sequence (‘ijm’ in this case), and type them on a response box with the keyboard.

In the test block, the participants had five minutes to complete a maximum of 15 letter series. The participants were free to manage their time and spend as much time as they wanted in each trial (within the 5-minute limit). They were informed about the time remaining at the beginning of every trial. Letter series were presented in ascending order of difficulty. This task included two practice trials. The participants were informed if they were able to figure out the next three letters of the series at the end of each trial (a textbox with the correct response was also presented in the feedback canvas). At the end of each block, they were informed about the number of correct responses.

*Number series* (Thurstone, 1938)*.* This task can be viewed as the numeric counterpart of the letter series. The number series was used as a measure of numerical inductive reasoning. A series of digits (e.g., ‘3’, ‘10’, ’24’, ’45’, ’73’) that followed a logical rule was displayed in each trial — here, digits increased from left to right, and each new number was obtained by adding the number on its left with the next multiple of seven (e.g., 10 = 3 + (7*1); 24 = 10 + (7*2)). The participants needed to identify the logical rule, estimate the next digit(s) of the sequence (‘108’), and type their responses with the keyboard.

Fifteen number series were included in the test block. Like in the letter series, the participants had five minutes to complete as much series as they could — they could manage their time as they saw fit and spend as much time as they wanted in each trial (within the 5-minute limit). A canvas with the remaining time was presented at the beginning of each trial. The series was presented in ascending order of difficulty. Two practice trials were completed before the test block. At the end of each trial, the participants were told if their responses were correct or incorrect. They also received feedback about the correct response in each trial. At the end of each block, they were informed about the number of series in which they provided a correct response.

*Raven’s Advanced Progressive Matrices (RAPM)* (Raven et al., 1998). This task was used to evaluate figural inductive reasoning. In each trial of this task, the participants assessed a pattern of black and white figures arranged on a 3 × 3 schema where the bottom-right figure was missing. The figures established a relational pattern between them (from left to right and from top to bottom). The participants had to choose among eight alternatives the figure that completed the pattern presented on the 3 × 3 schema. The participants pressed the numeric keys from ‘1’ to ‘8’ to select the figure they thought completed the pattern.

The participants completed the 18 odd-numbered problems from set II of the RAPM in the test block. They were allowed 10 minutes to solve as many problems as possible — the participants were informed about the remaining time at the beginning of every trial. They could manage their time and spend as much time as they wanted in each trial (within the 10-minute limit). Trials were presented in ascending order of difficulty. Before the test block, the participants completed the first two even-numbered problems of set II of the RAPM as practice trials. At the end of each trial, the participants were informed if they were able to select the figure that completed the pattern. They were also informed about the correct figure that completed the pattern. At the end of each block, they were informed about the number of correct responses.

### Scoring

The raw scores of all complex spans reflect the proportion of correctly recalled trials in the memory portions of these tasks (e.g., the proportion of correctly recalled to-be-remembered letters in the reading and operation spans and the proportion of correctly recalled red squares in the symmetry span). This scoring method is designated as partial-credit load scoring in the context of the complex span paradigm (Conway et al., 2005). We used this method because in past studies partial-credit scores presented higher internal consistencies than absolute scores (proportion of trials in which all to-be-remembered items were recalled in the right order) (Conway et al., 2005; Redick et al., 2012). Additionally, as Redick et al. (2012) stated, absolute scoring methods discard information that can be used to get better estimates of individual differences between participants. In the memory updating task, 1 point was awarded for each correctly updated digit. Thus, the possible score for each trial ranged from 0 to 3. Raw scores in the n-back task were calculated by adding the number of correct responses in target trials^2^. The length of the last sequence that each participant was able to recall correctly was used as the raw score in the multimodal span. The number of correct responses was used as the raw score in the binding and maintenance task^2^ and the *Gf* tasks. All raw scores were normalized — raw scores were converted to a scale that ranged from 0.00 to 1.00 through min-max normalization (Gajera et al., 2016). All subsequent analyses were based on normalized scores.

### Power analysis

Proactive Monte Carlo simulations (Wolf et al., 2013) were conducted to estimate the minimum sample size required to calculate all proposed structural equation models (SEM) and confirmatory factor analyses (CFA). The Monte Carlo simulations were computed in RStudio (version 4.1.3) with the package “simsem” (version 0.5.16) (Pornprasertmanit et al., 2022).

In proactive Monte Carlo simulation, multiple simulated datasets with a specified sample size are generated from a population model with known parameter values — these values can be derived from previous research. Then, the parameters of interest and their respective standard errors are estimated for each simulated dataset based on the known population values. Parameter estimates and standard errors are then averaged over all the simulated datasets (Beaujean, 2014). Several statistics are calculated from the average estimated parameters. Some of these statistics (relative parameter estimate bias, relative standard error bias, coverage, and statistical power) are used to determine if the specified sample size is sufficient to reproduce the population values and to obtain statistically significant parameter estimates (Wolf et al., 2013).

We followed the procedure outlined by Muthén and Muthén (2002) to compute the Monte Carlo simulations. These authors suggested running two Monte Carlo simulations with different seeds for each model to ensure the stability of the results (the seed determines the starting point for the random draws of the simulated datasets). 10,000 samples should be generated in each simulation. Muthén and Muthén (2002) also recommended some criteria to ensure that the chosen sample size is sufficient to achieve the desired statistical power and unbiased parameter estimates: relative bias and standard error bias must be ≤ .10 for all parameters — furthermore, relative standard error bias must be ≤ .05 for the parameters of interest. Coverage should range between .91 and .98, and statistical power should be at least .80.

We ran a proactive Monte Carlo simulation for every CFA and SEM conducted in our analysis. Parameter values used in the population model were taken from previous investigations. Parameter values for the reading span (.70), operation span (.66), n-back task (.55), binding and maintenance task (.86), letter series (.71), and number series (.70) were collected from the study of Wilhelm et al. (2013). The parameter value of the correlation between the WMC and the *Gf* factors (.83) was also derived from this study. Parameter values for the symmetry span (.73) and the RAPM (.76) were taken from the study of Kane et al. (2004), and the value for the memory updating task (.64) was obtained from the study of Schmiedek et al. (2009). We could not find a single investigation that used the multimodal span to assess the relationship between WMC and *Gf* or to derive a WMC factor. Thus, a value of .50 was attributed to this parameter (Katz, 2019). The results of the proactive Monte Carlo simulations suggested that a minimum sample size of 160 was necessary to compute the proposed SEM and CFA and to avoid a type I error (α = .05).

### Data treatment

Our initial database consisted of data from 169 participants. Data from five participants was dropped because they did not meet the inclusion criteria. Another participant was excluded from the analyses because he scored zero in more than one task.

Raw scores were converted to a scale that ranged from 0.00 to 1.00 through min-max normalization (Gajera et al., 2016). Then, the data were screened to detect univariate outliers with Microsoft Excel (version 2211). Any score that deviated more than 3 SD from the mean was considered a univariate outlier (Ang & Lee, 2010; Lewandowsky et al., 2010). Univariate outliers and zero scores were set to missing. A total of 33 scores (approximately 2% of the data) were set to missing.

Missing values were replaced by plausible values through multiple imputation. Multiple imputation generates several datasets (m) with slightly different estimates for missing values. Imputed values are predicted by a set of regression equations derived from the other observed variables included in the model plus a normally distributed residual term (Enders & Gottschall, 2011). A simulation study carried out by Graham et al. (2007) provided some guidelines about the number of datasets that need to be generated to calculate reliable estimates for missing data. The fraction of missing information (*γ*) in the original data influences the number of datasets that need to be generated. The smallest *γ* tested by Graham et al. (2007) was .10. These authors recommend using an m = 20 when this amount of missing data is present. Considering that the highest *γ* among our observed variables was equal to .07 (for the n-back task), we used multiple imputation to generate 20 datasets and replace the 33 missing values in our database. Imputed databases were generated in RStudio with the “mice” package (version 3.15.0) (Van Buuren & Groothuis-Oudshoorn, 2011). Missing values in WM tasks were exclusively derived from scores in other WM tasks, and missing values in reasoning tasks were solely derived from scores in other reasoning tasks. Additionally, observed values in the n-back task and the multimodal span were not included in the regression equations used to estimate the imputed values for each of these tasks because their scores presented a low and non-significant correlation (*r* = .14, *p* = .09). All analyses presented from this point forward were either based on these 20 datasets or pooled estimates of their data based on Rubin’s (1987) rules.

Next, we estimated Mahalanobis distances with the R package “stats” (version 4.1.3) (R Core Team, 2013) to assess the presence of multivariate outliers. The scores from one participant deviated significantly from the distribution (*p* < .001) and were excluded from further analysis.

At last, we evaluated the existence of univariate and multivariate normality in each of the 20 imputed datasets. The cutoffs suggested by Kline (2015) were considered to assess univariate normality in the scores of each task. According to this author, skewness indexes below 2 and kurtosis values under 4 indicate normal distributions. The amount of skewness and kurtosis inherent in the distribution of all variables in each of the 20 datasets was smaller than these values, suggesting the presence of univariate normality. Pooled estimates of skewness and kurtosis for each observed variable are displayed in Table 1. Mardia’s coefficients were computed to assess multivariate kurtosis in each of the 20 datasets. All coefficients were smaller than 3 and non-significant (*p* > .05), which was indicative of multivariate normal distributions (Romeu & Ozturk, 1993).

**Table 1:** Descriptive statistics and reliability estimates of the WM and Gf measures

Tasks	Mean	SD	Range	Skewness	Kurtosis	*α*	*ω*
RS	.76	.16	.27 – 1.00	-0.98	0.47	.92	.92
OS	.76	.15	.33 – 1.00	-0.50	-0.30	.91	.90
SS	.65	.17	.23 – 1.00	-0.24	-0.38	.90	.89
NB	.63	.24	.08 – 1.00	-0.24	-0.82	.82	.82
UT	.56	.27	.06 – 1.00	0.04	-1.29	.93	.93
MS	.38	.07	.27 - .55	0.33	-0.23	.49	.53
BT	.81	.15	.31 – 1.00	-0.85	0.17	.77	.77
LS	.38	.14	.07 - .73	0.19	0.03	.69	.74
NS	.41	.11	.13 - .80	0.67	0.57	.66	.72
RAPM	.48	.16	.06 - .89	0.13	-0.23	.65	.65

N = 162; WM, working memory; G*f*, fluid intelligence; SD, standard deviation; *α*, Cronbach’s alpha; *ω*, McDonald’s omega; RS, reading span; OS, operation span; SS, symmetry span; NB, n-back task; UT, memory updating task; MS, multimodal span; BT, binding and maintenance task; LS, letter series; NS, number series; RAPM, Raven's Advanced Progressive Matrices

### Statistical analysis

Descriptive statistics, reliability estimates, correlational analyses, exploratory factorial analysis (EFA), CFA, and SEM were computed in R with the packages “psych” (version 2.2.3) (Revelle, 2022), “miceadds” (version 3.15.21) (Robitzsch & Grund, 2022), “stats” (version 4.1.3) (R Core Team, 2013), “semTools” (version 0.5.6) (Jorgensen et al., 2022), and “semPlot” (version 1.1.6) (Epskamp, 2022).

*α* and *ω* were calculated for each task to assess their internal reliability. *α* and *ω* were computed at the level of individual trials. In both EFA and CFA, factors were extracted with maximum likelihood (ML) and an oblique rotation (promax) because our data was continuous and normally distributed (Fabrigar et al., 1999; Kline, 2015). Rubin’s rules (1987) were used to pool average parameter estimates and standard errors across the 20 imputed datasets.

Prior to the factor analysis, we computed the Kaiser-Meyer-Olkin measure (KMO) and Bartlett's test of sphericity to assess if our data was adequate for EFA. A KMO above .50 (Kaiser & Rice, 1974) and a significant Bartlett's test of sphericity value (Field, 2017) suggest that the characteristics of the data are adequate for factor analysis. A scree test (Zwick & Velicer, 1986) and a parallel analysis (Hayton et al., 2004) were conducted to evaluate how many factors should be extracted. We also applied Kaiser’s criterion (1970) to decide how many factors to retain (eigenvalues > 1).

For each CFA and SEM, standardized parameter estimates, squared multiple correlations, and error terms are presented in graphic representations of the models. As recommended by various authors (Beaujean, 2014; Hu & Bentler, 1995; Kline, 2015), several fit statistics were used to assess the adequacy of each CFA and SEM. The value of the chi-square test (*χ*^*2*^) is considered a key statistic in the assessment of model fit. This statistic reflects how similar the model-implied and the observed covariance matrix are. Higher *χ*^*2*^ values reflect larger differences between the model-implied and the observed covariance matrices. However, it is not recommended to rely solely on the value of the *χ*^*2*^ test to assess model fit because this test is sensitive to sample size (slight differences between the model-implied and the observed covariance matrices may lead to a significant *χ*^*2*^ value in models with moderate-to-high sample sizes) (Byrne, 2001). One way to bypass this issue is to divide the *χ*^*2*^ value by the degrees of freedom (*χ*^*2*^/df). Values smaller than 3 indicate an acceptable fit (Kline, 2015). We also report the comparative fit index (CFI) because this measure is less sensitive to sample size. The CFI is an incremental fit index that compares how much better the fit of the test model fares against an independent model with no correlations between the manifest variables. CFI values close to 0.95 suggest a good model fit (Hu & Bentler, 1999). Values above .90 are considered acceptable by some authors (Marsh et al., 2004). The root mean square error of approximation (RMSEA) estimates the difference between the model-implied and the observed covariance matrices per degree of freedom. RMSEA values smaller than 0.05 are considered excellent, values ranging from 0.05 to 0.08 are deemed acceptable, and values between .08 and .10 are regarded as mediocre (MacCallum et al., 1996). The standardized root mean square residual (SRMR) reflects the average of the standardized residuals between the model-implied and the observed covariance matrices. SRMR values smaller than 0.08 suggest a good model fit (Hu & Bentler, 1995).

## Results

### Descriptive statistics, reliability estimates, and correlations

Descriptive statistics for each WM and *Gf* task are presented in Table 1. Quartile, tercile, and median-based percentiles for the WM tasks are presented in Table 2. Descriptive statistics and percentiles were computed by pooling the values of the 20 datasets generated through multiple imputation.

**Table 2:** Percentiles for the WM tasks

Percentile	RS	OS	SS	NB	UT	MS	BT
5%	.40	.48	.35	.25	.14	.27	.50
25%	.67	.67	.55	.42	.33	.36	.70
33%	.75	.70	.58	.50	.39	.36	.75
50%	.78	.78	.66	.67	.53	.36	.88
66%	.85	.84	.73	.75	.72	.36	.88
75%	.88	.88	.75	.83	.81	.45	.94
95%	.97	.97	.92	1.00	.97	.54	1.00

N = 162; WM, working memory; RS, reading span; OS, operation span; SS, symmetry span; NB, n-back task; UT, memory updating task; MS, multimodal span; BT, binding and maintenance task

Table 1 also displays *α* and *ω* values for each task. Reliability was acceptable for all WM tasks except for the multimodal span (*α* = .49; *ω* = .53). The low reliability estimates of this task may be explained by its structure which was unique among the paradigms administrated in this study — the multimodal span was interrupted when the participants were not able to replicate two consecutive sequences with a given length. Furthermore, scores on this task were by far the lowest among the WM tasks (scores obtained in this task ranged from .27 to .54). This means that the participants were only able to replicate a maximum of four sequences. Thus, the reliability estimates of the multimodal span were calculated based on a small number of items (four trials), which may explain their low values (Tavakol & Dennick, 2011). Considering this, the results concerning this task should be interpreted with caution. However, we decided to retain the multimodal span in subsequent analyses because the magnitude of its zero-order correlations with other WM tasks was similar to the correlations between the binding and maintenance task and the other WM tests (Kane et al., 2004). Given their similar configurations, we hypothesize that both tasks measure the ability to bind characteristics of information to form new structures.

Additionally, some reliability estimates of the *Gf* tasks did not achieve the conventional thresholds for acceptable reliability — *α* and *ω* values ≥ .70 suggest acceptable internal consistency (Adadan & Savasci, 2012; McDonald, 1999). The *α* and *ω* values of the *Gf* tasks ranged from .65 to .74. However, the letter and number series and the RAPM are established *Gf* measures. Many studies have applied these tasks and obtained high reliability estimates (Buehner et al., 2005; Wiley et al., 2011). We believe that the relatively low reliability estimates found in this study are justified by the time restrictions imposed on these tasks — participants had five or 10 minutes to solve as many problems as possible. Similar *α* and *ω* values were found in studies that imposed analogous time constraints in these tasks (Wilhelm et al., 2013).

The correlation matrix of all WM tasks is presented in Table 3. The matrix was also computed by pooling the values of the 20 datasets generated through multiple imputation. All values were positive (most *r*s > .30) and significant, except for the correlation between the n-back task and the multimodal span. The magnitude of the correlations was similar to those verified in previous studies (Lewandowsky et al., 2010; Oswald et al., 2015; Schmiedek et al., 2014). The lack of a significant correlation between the n-back task and the multimodal span was not regarded as particularly problematic because we considered that the n-back task and the multimodal span measure different specific aspects of WM. The moderate magnitudes of the correlations were adequate for CFA and SEM because they were not high enough to suggest the presence of multicollinearity (*R*^*2*^ > .90) (Kline, 2015) and not low enough to imply potentially spurious associations among the indicators (Kane et al., 2004).

**Table 3:** Correlation matrix of the WM tasks

Task	RS	OS	SS	NB	UT	MS	BT
RS	–						
OS	.53^**^	–					
SS	.52^**^	.41^**^	–				
NB	.35^**^	.35^**^	.27^**^	–			
UT	.48^**^	.56^**^	.54^**^	.37^**^	–		
MS	.22^*^	.26^*^	.30^**^	.14	.39^**^	–	
BT	.27^*^	.24^*^	.33^**^	.33^**^	.30^**^	.28^**^	–

N = 162; WM, working memory; RS, reading span; OS, operation span; SS, symmetry span; NB, n-back task; UT, memory updating task; MS, multimodal span; BT, binding and maintenance task

^*^ *p* < .01. ^**^ *p* < .001

### Factor analyses

An EFA was computed to freely evaluate the underlying structure of the WM tasks. The KMO (.83) (Kaiser & Rice, 1974) and Bartlett’s test of sphericity (*χ*^*2*^(21) = 301.53, *p* < .001) suggested that our data was adequate to conduct this analysis. The results of the scree test, the parallel analysis, and Kaiser’s criterion (1970) suggested that a single factor was enough to accommodate all WM tasks (eigenvalue = 2.64), which is indicative of convergent validity. This factor accounted for 38% of the variance in the WM measures. This corresponds to a large effect size (ƒ^2^ = .52). All tasks presented acceptable factor loadings (> .40) (Field, 2017). We interpreted this factor as an indirect estimate of WMC.

**Fig. 2:**
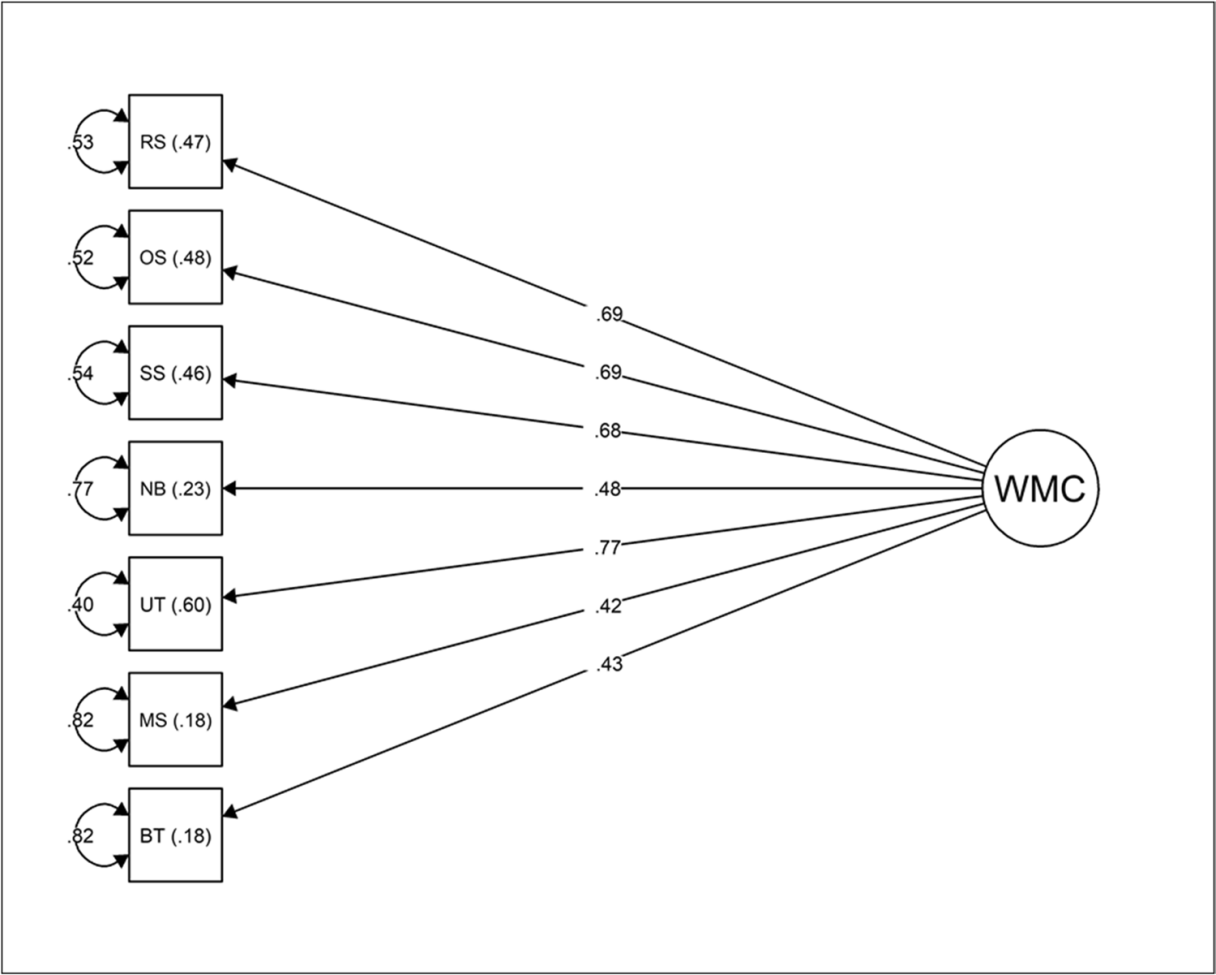
Model 1: CFA for the unifactorial model of WMC. Circles represent latent factors. Rectangles represent manifest variables. Curved arrows represent standardized error terms. RS, reading span; OS, operation span; SS, symmetry span; NB, n-back task; UT, memory updating task; MS, multimodal span; BT, binding and maintenance task; WMC, working memory capacity

Then, a CFA was computed to confirm that the general WMC factor extracted in the EFA presented an adequate structure to accommodate all seven tasks included in the battery. The loadings of all WM tasks were freely estimated (Beaujean, 2014). Model 1 provided a good fit to the data, *χ*^*2*^(14) = 22.00, *p* = .08; *χ*^*2*^*:df* = 1.57; CFI = 0.97; RMSEA = 0.059; SRMR = 0.046. All factor loadings were acceptable and significant (*p* > .001), which indicated high factor-based reliability. Standardized factor loadings, squared multiple correlations, and standardized error terms are presented in Fig. 2. Additionally, Model 1 presented good reliability (*ω* = .80). The examination of the standardized residual matrix of covariances revealed no significant residual values. Thus, no *post hoc* modifications were applied to the model.

A SEM was computed to assess the predictive validity of the battery. *Gf* measures have been widely used as a criterion to validate WM tasks because WM is considered one of the main predictors of *Gf* (if not the major one) (Kane et al., 2004; Oswald et al., 2015; Wilhelm et al., 2013). Thus, we extended model 1 to include a *Gf* factor. This factor was derived from three reasoning tasks that covered the verbal, numeric, and visuospatial domains typically attributed to *Gf* (Unsworth et al., 2009).

**Fig. 3:**
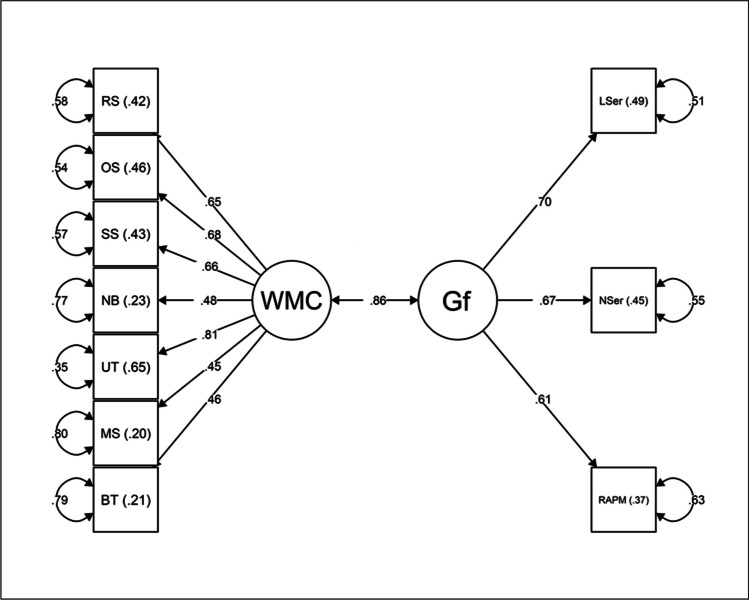
Model 2: SEM assessing the relationship between WMC and *Gf*. Circles represent latent factors. Rectangles represent manifest variables. Curved arrows represent standardized error terms. RS, reading span; OS, operation span; SS, symmetry span; NB, n-back task; UT, memory updating task; MS, multimodal span; BT, binding and maintenance task; WMC, working memory capacity; *Gf*, fluid intelligence; LSer, letter series; NSer, number series; RAPM, Raven’s Advanced Progressive Matrices

The correlation between the WMC and *Gf* factors was estimated. The loadings of the reasoning tasks on the *Gf* factor were freely estimated (Beaujean, 2014). The chi-square test associated with Model 2 was significant (*χ*^*2*^(34) = 64.95, *p* = .001). However, as previously stated, significant χ^2^ values are not uncommon for moderate-to-larger sample sizes because this test is sensitive to sample size (Kline, 2015). Due to this, the *χ*^*2*^/df is considered a more appropriate measure to assess model fit. Values smaller than 3 are considered acceptable. Model 2 presented a *χ*^*2*^/df of 1.94, which indicated an acceptable model fit. Additionally, all alternative fit indexes suggested that Model 2 provided an acceptable fit to the data: CFI = .93; RMSEA = 0.075; SRMR = 0.054. All factor loadings were significant (*p* > .001), which implied high factor-based reliability. The WMC factor was largely correlated with the *Gf* factor (*r* = .86, *p* < .001). The magnitude of this correlation was similar to those found in other studies (Hicks et al., 2016; Schmiedek et al., 2009, 2014; Wilhelm et al., 2013). Standardized factor loadings, squared multiple correlations, and standardized error terms are presented in Fig. 3. The *Gf* factor presented good reliability (*ω* = .70). The examination of the standardized residual matrix of covariances revealed significant residual values between the scores of the memory updating task and the number series (3.17) and the scores of the number series and the RAPM (-2.88) — values > 2.58 are considered large (Byrne, 2001). Despite the discrepancy between observed and expected values, no post-hoc modifications were applied to Model 2 because we wanted the WM indicators to behave in the same way as they did in Model 1 — the main purpose of this model was to assess the external validity of the unifactorial model of WMC. Additionally, Model 2 had acceptable fit values.

### Using single tasks, homogenous factors, and heterogenous factors to estimate WMC

The importance of employing several paradigms to get an accurate measurement of WMC is an issue that has been regularly highlighted in WMC research (Ecker et al., 2010; Kane et al., 2004; Wilhelm et al., 2013). However, WMC has been gauged with a single task (Monk et al., 1997; Xie et al., 2020) or several tasks from the same paradigm (Burgoyne et al., 2023; Felez-Nobrega et al., 2018) in several studies. Using a single task or paradigm to assess WMC can lead to contamination due to task-specific or paradigm-specific variance. Considering this, several authors have suggested that it is important to employ multiple paradigms that assess different functional aspects of WM to get a precise evaluation of WMC (Lewandowsky et al., 2010; Schmiedek et al., 2014; Wilhelm et al., 2013). However, to our knowledge, only a single study (Schmiedek et al., 2014) tried to assess how different combinations of heterogeneous sets of WMC paradigms fare against WMC factors exclusively composed of tasks from a single paradigm. The results of this investigation suggested that heterogenous triplets of WMC tasks correlated more strongly with a reasoning factor than combinations of three tasks from the same paradigms (e.g., updating tasks).

We conducted a similar analysis to assess if heterogeneous sets of WMC tasks provide a better estimate of WMC than groups of tasks from the same paradigm or single tasks. In this analysis, we estimated the amount of unique variation shared between a *Gf* factor — a construct highly related to WMC (Kane et al., 2004; Oswald et al., 2015; Wilhelm et al., 2013) — and (1) each WM task individually, (2) homogenous latent factors based on tasks from the same paradigm (complex spans, updating tasks, or binding tasks), and (3) heterogeneous latent factors extracted from all possible combinations of triplets that included a task from each of the three paradigms included in our battery.

We computed seven SEM in which WMC was represented by a single task, three SEM in which the WMC factor was derived from a single paradigm (complex spans, updating tasks, or binding tasks), and twelve SEMs in which the WMC factor was computed based on three WM tasks from different paradigms (e.g., operation span, n-back task, and binding and maintenance task). In models that only included updating and binding tasks, the loadings of the WM tasks were set to equal because the measurement model of WMC only included two indicators (e.g., memory updating task and n-back task) (Beaujean, 2014). In SEM models in which WMC was represented by a single task no latent WMC factor was created. We assessed the amount of unique variance shared between the single WM task and the latent *Gf* factor (Muthén & Muthén, 2003). The fit indices of all models estimated in the permutation analysis are presented in Table 4. These values are ranked according to the amount of variance shared between the WMC and the *Gf* factors — however, poorly fitted models are presented at the bottom of the table. All factor loadings from every model were significant (*p* < .001).

**Table 4:** Variance shared between the WMC and Gf factors and fit indexes for all models included in the permutation analysis

Indicators	*R*^*2*^	*χ*^*2*^	*p*	*χ*^*2*^:*df*	CFI	RMSEA	SRMR
RS-UT-BT (HeF)	.90	15.33	.050	1.92	0.97	0.07	0.04
OS-UT-BT (HeF)	.83	19.38	.010	2.42	0.95	0.09	0.05
SS-NB-MS (HeF)	.79	14.20	.080	1.78	0.96	0.07	0.04
RS-UT-MS (HeF)	.78	17.33	.030	2.17	0.96	0.08	0.04
OS-NB-MS (HeF)	.77	16.06	.040	2.01	0.95	0.08	0.04
RS-NB-MS (HeF)	.75	12.74	.120	1.59	0.97	0.06	0.04
OS-NB-BT (HeF)	.75	14.60	.070	1.83	0.96	0.07	0.04
OS-UT-MS (HeF)	.75	19.80	.010	2.48	0.95	0.10	0.05
SS-NB-BT (HeF)	.68	10.31	.240	1.29	0.99	0.04	0.04
RS-NB-BT (HeF)	.65	8.65	.370	1.08	1.00	0.02	0.04
RS-OS-SS (HoF)	.52	19.06	.010	2.38	0.95	0.09	0.05
BT (ST)	.23	0.81	.670	0.41	1.00	0.00	0.01
RS (ST)	.22	4.55	.100	2.28	0.98	0.09	0.03
MS (ST)	.19	0.69	.710	0.35	1.00	0.00	0.01
NB (ST)	.14	2.26	.320	1.13	1.00	0.03	0.03
MS-BT (HoF)	.64	10.37	.070	2.07	0.96	0.08	0.09
SS-UT-MS (HeF)	.73	25.84	< .001	3.23	0.93	0.12	0.05
SS-UT-BT (HeF)	.81	28.23	< .001	3.53	0.92	0.12	0.05
OS (ST)	.29	8.70	.010	4.35	0.94	0.14	0.04
UT (ST)	.55	9.29	.010	4.65	0.95	0.15	0.04
SS (ST)	.25	9.50	.010	4.75	0.94	0.15	0.04
NB-UT (HoF)	.89	28.44	< .001	5.69	0.87	0.17	0.12

N = 162; *R*^*2*^*,* amount of variance shared between the *Gf* factor and a WMC factor derived from the tasks displayed in the columns “Indicators”; *χ2*, chi-square statistic; *p*, p-value; *χ*^*2*^*:df*, chi square-to degrees of freedom ratio; CFI, comparative fit index; RMSEA, root mean square error of approximation; SRMR, standardized root mean squared residual; HeF, heterogeneous latent factor; HoF, homogenous latent factor; ST, single task; RS, reading span; OS, operation span; SS, symmetry span; NB, n-back task; UT, memory updating task; MS, multimodal span; BT, binding and maintenance task

The results of this analysis suggested that heterogeneous factors provided better estimates of WMC than homogenous factors or single tasks. All acceptable heterogenous WMC factors shared more variance with the *Gf* factor than any single task or the only acceptable WMC factor derived from a single paradigm — the acceptable heterogenous WMC factor that shared the least variance with the *Gf* factor was still able to account for more variance (65%) than the only acceptable homogenous WMC factor (52%) or the most valid single WM task (23%).

## Discussion

The main purpose of this study was to assess the psychometric properties of the OpenWMB, an open-source and automated battery of heterogenous WM tasks for OpenSesame. The battery was validated in an experiment involving a sample of Portuguese citizens, aged between 18 and 35 years, who completed at least a high school degree. We provided detailed descriptions of the performance of the participants on all seven WM tasks included in our battery (we presented measures of central tendency, variability, and percentiles) because there is a limited amount of data available regarding the WMC of the Portuguese population. To our knowledge, only one study used one of the tasks included in our battery (the reading span) to assess the WMC of a sample of Portuguese adults (Gaspar & Pinto, 2001). Thus, the data reported in this article provides one of the few accounts of the WMC of Portuguese citizens.

The battery as a whole and the great majority of the WM tasks included in this instrument presented good internal consistency (Adadan & Savasci, 2012; McDonald, 1999). Based on previous research, these values were within the expected range (Lewandowsky et al., 2010; Oswald et al., 2015; Schmiedek et al., 2014; Wilhelm et al., 2013). The only exception to this trend was the multimodal span (*α* = .49; *ω* = .53). As we stated in the Results section, we believe that these values are explained by the small number of trials that the participants completed in this task (Tavakol & Dennick, 2011). The poor performance in this task may be explained by the fact that the multimodal span was interrupted when the participants were not able to replicate two consecutive sequences with a given length. However, we kept the multimodal span in the battery because its correlations with other WM tasks presented a magnitude similar to those of the binding and maintenance task (Kane et al., 2004). Additionally, the multimodal span presented an acceptable and significant loading in the WMC factor. Nonetheless, researchers who intend to use this task in future studies should interpret its results with caution.

The OpenWMB has an appropriate convergent validity: this was substantiated by the moderate to high positive correlations between the WM tasks (most *r*s > .30) and the results of the EFA and CFA. The magnitude of the correlations was similar to those verified in previous studies (Lewandowsky et al., 2010; Oswald et al., 2015; Schmiedek et al., 2014). All tasks loaded into a single latent factor that captured a common source of variance. The variance shared by the tasks was probably caused by individual differences in WMC. The single factor accounted for a substantial amount of variance in the WM tasks (38%), which denotes a large effect size (ƒ^2^ = .52).

We would like to highlight that both binding tasks loaded significantly in the WMC factor, suggesting that this paradigm is a valid method to measure WMC. To our knowledge, this is the first available battery to include binding tasks, even though these tasks loaded in the same latent factor as complex spans and updating tasks in several individual differences studies (Oberauer et al., 2003; Wilhelm et al., 2013).

The general WMC factor also presented a large and significant correlation with a latent factor derived from three *Gf* tasks (*r* = .86, *p* > .001). Thus, our results replicated the common finding that WMC and *Gf* are highly correlated, which indicates that our battery presented good predictive validity. Several studies found a correlation between *Gf* and WMC with a magnitude > .80 (Schmiedek et al., 2009, 2014; Wilhelm et al., 2013). However, the size of this correlation is relatively larger than the ones found in other studies (Engle et al., 1999; Felez-Nobrega et al., 2018; Kane et al., 2004; Oswald et al., 2015) — the correlations between WMC and *Gf* ranged from .47 to 0.69 in these investigations. The different magnitudes of these correlations may be explained by the distinct methods used to derive the WMC factor in these studies. Similarly to Schmiedek et al. (2009, 2014) and Wilhelm et al. (2013), we extracted our WMC factor from multiple tasks from different paradigms. On the contrary, Engle et al. (1999), Felez-Nobrega et al. (2018), Kane et al. (2004), and Oswald et al. (2015) used tasks from the same paradigm (complex spans) to compute their WMC factors. Thus, these results provide some support to the argument that using several tasks from different paradigms leads to better WMC estimates (Foster et al., 2015; Wilhelm et al., 2013).

To get a more systematic view of the best method to estimate WMC, we performed a permutation analysis in which we calculated the amount of variance shared between the *Gf* factor and (1) each WM task individually, (2) homogenous latent factors exclusively based on tasks from the same paradigm (complex spans, updating tasks, or binding tasks), and (3) heterogeneous latent factors extracted from all possible combinations of triplets that included a task from each of the three paradigms included in our battery. The results of this analysis suggested that heterogenous WMC factors provided the best estimates of WMC (see Table 4), which replicated the findings of Schmiedek et al. (2014). All acceptable heterogeneous factors shared more variance with the *Gf* factor than any single WM task or the only acceptable homogenous factor (which was derived from three complex spans). In fact, the gap between the heterogeneous factors and the other types of estimates was probably even wider than is suggested by the permutation analysis: in models derived from single tasks, the variance shared between the WMC indicator and *Gf* probability reflected a mixture of WMC and task-specific and paradigm-specific variance. This estimate probably involved an amount of paradigm-specific variance that was inseparable from WMC variance in models that included homogenous WMC factors.

The superiority of the WMC estimates derived from heterogenous factors may be explained by the inclusion of tasks with different structures that probably measured various functional aspects of WM — complex spans require simultaneous storage and processing of information (Redick et al., 2012; Unsworth et al., 2009) while updating tasks demand continuous refreshing of mental representations (Ecker et al., 2010), and binding tasks involve linking several characteristics of stimuli to form new structures and relationships (Oberauer et al., 2003; Wilhelm et al., 2013). Conversely, assessing WMC with a single task or a latent factor derived from tasks from the same paradigm probably neglects some functional aspects of WM which probably leads to a biased view of this construct. Our results suggested that, ideally, WMC estimations should be extracted from several tasks from multiple paradigms that tap into the different content domains and functional aspects ascribed to this system (Waris et al., 2017). This approach will significantly increase the amount of WMC variance captured by the model and partial out construct-irrelevant variance, which will reduce bias in the interpretation of the derived factor(s) (Oswald et al., 2015; Schmiedek et al., 2014).

We would like to stress that we derived the heterogeneous latent factors from three tasks because this structure contained the minimum number of indicators needed to represent all paradigms included in our battery in the measurement models of WMC. However, the acceptable WMC estimates obtained in the permutation analysis suggested that a small number of WM tasks is enough to get a valid estimate of WMC with a reduced time cost. This approach may be particularly useful for investigations with a limited testing time that cannot administer a set of tasks that covers the full range of functional aspects and content domains of WMC.

Before we conclude this section, we would like to discuss some limitations of our work and provide suggestions for future research.

First, we only assessed the psychometric proprieties of the Portuguese version of the battery. Another validation study should be conducted for the English version of the OpenWMB to evaluate if the battery is a psychometrically valid method to assess WMC in English-speaking populations.

Second, we only administrated the OpenWMB once with a single sample. Thus, we did not evaluate retest effects. It would be interesting to assess if our battery is permeable to this phenomenon, as some studies suggested that WM tasks are prone to retest effects (Scharfen et al., 2018). In the future, the battery should be administered multiple times to samples with different characteristics to evaluate if the psychometric properties of the instrument are constant across multiple administrations and various samples.

Third, unlike other automated instruments (Oswald et al., 2014; Redick et al., 2012; Stone & Towse, 2015), our battery does not randomize the order of blocks with different set sizes in the complex spans. Thus, the participants involved in the validation study could anticipate the number of stimuli that would be presented in each block. This may have led to the development of rehearsing strategies that may have introduced some confounding variance in the WMC estimate. Randomizing the number of stimuli presented in each block could have helped to curtail such strategies (Unsworth et al., 2005).

Fourth, in the permutation analysis, the models with factors exclusively composed of updating and binding tasks (models NB-UT (HoF) and MS-BT (HoF) from Table 4) were extracted from two tasks. Even though it is possible to derive a latent factor from two indicators, these models tend to be more problematic as this method requires constraining the factor loadings of both indicators to equal to get a just-identified model. This is why most authors recommend deriving latent factors from at least three indicators (Beaujean, 2014; Kline, 2015). Thus, the aforementioned models were probably not the best WMC estimates exclusively derived from updating and binding tasks. Future studies should attempt to replicate this analysis using a higher number of updating and binding tasks as indicators of WMC and evaluate how they fare against WMC factors extracted from tasks from distinct paradigms.

The last limitation that we would like to discuss concerns a possible flaw in the design of our battery. As we previously stated, we included three complex spans because previous studies suggested that a non-negligible portion of the variance in these tasks is content-specific (Kane et al., 2004; Oberauer et al., 2000). Thus, we included a verbal, a numeric, and a spatial complex span to partial out domain-specific variance from our WMC estimates. On the other hand, we only selected two updating tasks and two binding tasks because most authors suggested that the ability to continuously update mental representations and the capacity to bind characteristics of information to form new structures are domain-general (Baddeley, 2000; Oberauer et al., 2003; Waris et al., 2017) — although this premise is not universally accepted (Nee et al., 2013). However, only including two updating and two binding tasks in our battery may have hampered the emergence of a second line of latent factors that could reflect some of the functional aspects of WM (simultaneous storage and processing, updating, and binding) in the EFA and subsequent CFA. Future studies should attempt to replicate our study with a larger number of tasks per paradigm and assess if it leads to an acceptable WMC model that can identify some of the functional aspects attributed to WMC by other authors (Oberauer et al., 2000, 2003).

To recap and conclude, various preeminent automated batteries of WM tasks have been developed in the past years (Foster et al., 2015; Lewandowsky et al., 2010; Ma et al., 2017; Oswald et al., 2015; Stone & Towse, 2015; Unsworth et al., 2005, 2009). However, some of these instruments were programmed on platforms that require the purchase of a commercial license, while others only included a single class of WM tasks. Furthermore, these batteries are still only available in certain languages — none of these instruments includes a Portuguese version. To address these issues, we programmed the OpenWMB, an automated battery that includes seven WM tasks from three distinct paradigms (complex spans, updating tasks, and binding tasks). The OpenWMB is available in Portuguese and English. The tool runs on an open-source platform — OpenSesame (Mathôt et al., 2012) — and is freely available online in a ready-to-download format.

The OpenWMB presented good psychometric properties (internal consistency, convergent validity, and predictive validity). Additionally, it produces a reliable and valid general estimate of WMC by tapping both into its functional aspects (e.g., simultaneous storage and processing, updating, and binding abilities) and content domains (verbal, numeric, and spatial) while reducing paradigm-specific and task-specific variance.

The battery possesses some flexible features that can be implemented without any programming knowledge (e.g., users can choose to only administrate a portion of the tasks) and includes a data processing script that converts all data collected into an easily interpretable format that is ready for data analysis (in platforms like R or SPSS). In its current form, the OpenWMB only runs the WM tasks in a fixed format. However, because of its open-source nature (the battery and the processing script were programmed using Python and OpenSesame scripting), users can adapt our source code to create alternative versions of the WM tasks (e.g., augment the number of trials, remove practice blocks, create short versions of the tasks).

The battery is suitable for group testing, is entirely computer-paced, has embedded instructions for each task, and has automatic scoring. Thus, the OpenWMB can easily be adapted to the needs of different investigations (e.g., researchers with limited testing time can choose to administrate a reduced version of the battery) and can be used in individual differences, experimental, and clinical studies.

## Appendix

### Using the OpenWMB

#### Download and tutorials

The battery can be downloaded from the GitHub repository associated with the webpage https://zenodo.org/doi/10.5281/zenodo.10600494 — again, to access this repository you will need to locate and click on the GitHub URL that is presented on the Zenodo page. This repository contains four folders with different versions of the battery (OpenWMB_EN_macOS.zip, OpenWMB_EN_Windows.zip, OpenWMB_PT_macOS.zip, and OpenWMB_PT_Windows.zip).

Each of these folders includes the “.osexp” file that runs the OpenWMB and a data-processing script named “Script_Organize_log_files.py”. They also hold several documents with tutorials and examples that explain how to download, install, and use the OpenWMB and how to analyze and interpret data collected with this instrument.

To learn how to properly download and use the OpenWMB, it is paramount to read the ‘README’ section of the repository that contains the battery (we recommend downloading the OpenWMB from the GitHub repository because the 'README' section is not displayed on the Zenodo page).

#### Requirements and installation

The OpenWMB runs on OpenSesame (Mathôt et al., 2012). Thus, our instrument requires the installation of this platform. The OpenWMB was programmed and tested on OpenSesame v3.3.11. However, the battery also works well with more recent versions of OpenSesame v3.3. Our program also requires the Mousetrap plugin developed by Kieslich and Henninger (2017). Both OpenSesame and Mousetrap are freely available online. Installation files and instructions can be found at https://osdoc.cogsci.nl/3‌.3/download/ and https://github.com/PascalKieslich/mousetrap-os, respectively. Because our instrument depends on OpenSesame and Mousetrap, the authors that intend to use the OpenWMB should also cite the articles of Kieslich and Henninger (2017) and Mathôt et al. (2012).

The OpenWMB works well with Microsoft Windows (Vista or above) and Apple macOS (the battery was tested in macOS Ventura, version 13.2.1). The .zip folders that contain the different versions of the OpenWMB (available on the webpage https://zenodo.org/doi/10.5281/zenodo.10600494 and on the corresponding GitHub repository) include an installation guide with further details.

#### Running the tasks

As stated in previous sections, the OpenWMB includes seven WM tasks (reading span, operation span, symmetry span, n-back task, memory updating task, binding and maintenance task, and multimodal span). The battery is suitable for group testing, is entirely computer-paced, has embedded instructions for each task, and has automatic scoring.

The order of presentation of the tasks is counterbalanced by an algorithm that applies a Latin square design which ensures that all tasks are presented an equal number of times in each position across participants (on complete administrations). The participant number determines the order of presentation in each administration of the OpenWMB — OpenSesame will request the participant number when the battery is launched. The counterbalancing algorithm continuously subtracts seven from the participant number until the resulting digit is between 1 and 7. Then, this number is divided by 7. The fractional portion of the division determines the order of presentation of the tasks. All possible presentation orders are described on page two of the user guide.

When the participant number is selected, the battery will request the location where the file with collected data should be stored. The main folder of the OpenWMB includes a sub-folder entitled “data_participants”. It is paramount to store all data files in this sub-folder. The OpenWMB includes a data processing script called “Script_Organize‌_Log‌_Files.py”. This script will only function properly if the data files are stored in the sub-folder “data_participants”.

At this point, the battery will be launched. The first menu lets the users choose which tasks they want to use. By default, the program administrates the seven tasks included in the instrument and a brief sociodemographic questionnaire. However, it is possible to only apply a portion of the tasks. To deactivate one or more tasks, the users simply need to change the tick in the homonymous checkbox(s) to “No” in the first menu of the battery. The sociodemographic questionnaire can also be deactivated. Thus, the OpenWMB can be used in studies that want to measure WMC with a single task (e.g., operation span), for example. In this case, the users simply need to deactivate all other tasks. By the same standard, the OpenWMB can also be applied in investigations that only want to employ a single task paradigm (e.g., complex span) and studies that wish to use a small set of tasks from different WM paradigms (e.g., operation span, memory updating task, and binding and maintenance task).

After task selection, a canvas with the general instructions of the battery will appear on the screen. When this canvas is presented, the users/researchers can start to administrate the battery to their participants. The battery is entirely automated and contains detailed instructions for each task. Thus, the participants should be able to complete the battery independently from this point forward.

The OpenWMB includes a user guide that explains all these features in detail. In its current form, the OpenWMB only runs the WM tasks in a fixed format. However, because of its open-source nature, users with experience with Python and OpenSesame scripting can modify the source code of the .osexp file that contains the battery and the Python data processing script to create alternative versions of the WM tasks (e.g., augment the number of trials, remove practice blocks, create short versions of the tasks).

#### Data processing

As previously stated, the OpenWMB includes a data processing script entitled “Script_Organize_Log_Files.py”. This script was programmed in Python and converts data collected by the battery in an easily interpretable format that is ready for data analysis (in platforms like R or SPSS). This script also normalizes the raw scores of the participants in each WM task and calculates some descriptive statistics (e.g., mean, standard deviation) for these scores and the sociodemographic variables. This script will only work properly if the data files are stored in the sub-folder “data_participants”.

To run this script, the users need to double-click on it. This will open Rapunzel (a code editor that runs Python scripts) (Mathôt, 2023). To run the data processing script, the users simply need to click on the ‘Run project or file’ button of Rapunzel. A detailed set of instructions explaining how to use the data processing script is available in section II of the user guide of the OpenWMB.

When the script is executed, an Excel file called “BD_WM_Battery” will appear in the main folder of the battery. This file contains several sheets. The first sheets present the same name as the administered WM tasks (e.g., ‘symmetry span’, ’n-back task’, ‘binding and maintenance task’). Each sheet contains important information regarding the corresponding WM task (e.g., score, RT, stimuli displayed, type of condition). A detailed description of the data stored in relevant columns of each sheet is presented in the homonymous notepad that can be found in the sub-folder “interpret_loggers”. The file “BD_‌WM_‌Battery” also includes sheets with the raw and normalized scores of the participants in every WM task that was administered. The latter contains the scores of the participants in each WM task on a scale ranging from 0.00 to 1.00. The sheets with raw and normalized scores can be directly imported into statistical analysis software such as R or SPSS. The last two sheets of the file include descriptive statistics (e.g., mean, standard deviation, minimum, maximum, and quartiles) for the raw and normalized scores of each WM task and the sociodemographic variables.

#### English version

The Zenodo record located at https://zenodo.org/doi/10.5281/zenodo.10600494 and the corresponding GitHub repository also includes English versions of the OpenWMB that run on Windows and macOS operating systems. These versions are equal to their Portuguese counterparts in all accounts, except for the instructions presented at the beginning of each task. Additionally, the English version does not include the reading span (because the sentences for this task were formulated in Portuguese).

However, we would like to stress that no validation study was conducted for the English version of the battery — all analyses presented in this article were conducted with a Portuguese sample and only concerned the Portuguese version of the battery. Furthermore, the English version of the OpenWMB was translated by the authors of this manuscript (all native Portuguese speakers). No forward or backward translation was carried out (Fenn & Hambrick, 2012).

We translated the battery to English because the Portuguese and the English languages share the same alphanumeric characters and the stimuli used on all the alphanumeric tasks, apart from the reading span, were single letters or numbers. Thus, the other six WM tasks required no translation besides the initial instructions.

Above all else, we decided to offer an English version of the OpenWMB because these paradigms are universally used to measure WMC. We believe that a free English battery of WM tasks may help researchers who want to evaluate WMC in English-speaking populations by offering them a broad, flexible, and fully automated range of WM paradigms. However, the researchers who decide to use the English version of the battery should consider the caveats presented in this section.

Any researchers who wish to validate the English version of the battery or help to improve its translation are invited to contact the authors of this manuscript. Researchers who use the English version of the OpenWMB should also cite this article.

## Footnotes

1 In this article we use the term “trial” to describe a single instance in which a stimulus is presented and processed according to the requirements of the task (e.g., in the reading span a trial comprises the presentation of a sentence, the assessment of the presence of a syntactical error, and the presentation and memorization of the to-be-remembered letter). We use the term “block” to define a set of trials.

2 The OpenWMB also computes alternative raw scores for the n-back task and the binding and maintenance task. The alternative raw score of the n-back task is estimated by adding the number of correct responses in target and non-target trials. The alternative raw scores of the binding and maintenance task are based on the ‘accuracy score’ and the ‘processing score’ proposed by Quinette et al. (2006). However, all analyses presented in this article were computed with estimates based on the scoring procedures presented in the “scoring” subsection.
